# AI assisted triage of UK patients in mental health care services: a qualitative focus group study of patients’ attitudes

**DOI:** 10.1186/s12888-025-07329-7

**Published:** 2026-01-13

**Authors:** Katharine A. Smith, Julia Hamer-Hunt, Andrey Kormilitzin, Helen Page, Dan W. Joyce, Andrea Cipriani, the CHRONOSIG group*, Jane Grieve, Oana Colta, Jim McMahon, Lindsey Hammil-Evans, Colin W. Larkworthy, Alison Ali Bryant, Maxine McCarthy, Nathan Davies, K. M. Keates, Julie Blaney, Sean Barnes, Terry Bryant, Saiqa Ahmed, Joanna C, Katie Brunwin, Omar Laftah

**Affiliations:** 1 Department of Psychiatry, University of Oxford, Oxford, UK; 2 Oxford Health NHS Foundation Trust, Warneford Hospital, Oxford, UK; 3 Oxford Precision Psychiatry Lab, NIHR Oxford Health Biomedical Research Centre, Oxford, UK; 4 Department of Primary Care and Mental Health, Institute of Population Health, University of Liverpool, Liverpool, UK; 5 Mersey Care NHS Foundation Trust, Kings Business Park, Trust Offices/V7 Buildings, Prescot, L34 1PJ UK; 6 Mental Health Research for Innovation Centre (M-RIC) and the Civic Health Innovation Labs (CHIL), Liverpool Science Park, 131 Mount Pleasant, Liverpool, L3 5TF UK

**Keywords:** Depression, Triage, AI, Clinical decision making, Electronic health record, Qualitative study

## Abstract

**Background:**

The referral process between healthcare services can be complex, especially in psychiatry, leading to significant delays and ‘hidden waiting lists’. Digital approaches may be helpful. The CHRONOSIG (CHRONOlogical SIGnature) project aims to improve the referral and triage process by applying machine learning (ML) technology to information in electronic health records. We used a focus group methodology to ascertain the views of patients and participants on using CHRONOSIG and similar digital approaches to support decision making in triaging referrals in difficult to treat depression, and the potential benefits and disadvantages of such an approach.

**Methods:**

A lived experience participant focus group (*N* = 16) was held on 25th September, 2024, with a lived experience chair. Participants were recruited by convenience sampling. Data were analysed thematically and managed using the Framework method, with double coding of transcripts, and reported using COREQ guidelines.

**Results:**

Main themes from the analysis were: (i) the complexity of mental health needs assessments; (ii) challenges in the current mental health system; (iii) general challenges of using a computer/artificial intelligence based tool for risk prediction and clinical decision support; (iv) differences and similarities in using a computer-based prediction tool in mental health vs. in physical health; (v) possible benefits and harms; (vi) factors to consider in the future.

**Conclusions:**

Patient engagement is a key challenge for digital tools in mental health, but previous studies in digital decision support tools have focussed on clinician feedback. In this study we ascertained the views of lived experience participants in mental healthcare triage and referral in difficult to treat depression. Participants identified delays, errors and confusion in the referral process and expressed positive views on the ability of the CHRONOSIG tool to help to improve waiting times and time spent between services, particularly when used as an addition to a high-quality clinical consultation. In many countries there are shortfalls in mental health care provision with increasing waits in both recorded and unrecorded waiting lists. This study supports a potential route to improve these processes; by more accurately and efficiently identifying the needs of patients and matching these to suitable services and research opportunities.

**Supplementary Information:**

The online version contains supplementary material available at 10.1186/s12888-025-07329-7.

## Background

In healthcare, patients often move between services using a process of referral from one clinical team, followed by assessment and triage by the accepting team. At each of these referral points, important-decision making occurs and transfer of accurate information is crucial. This is a particularly complex process in mental health, where the majority of information is contained in narrative formats, in referral letters and electronic health records (EHRs) describing the patient’s history and background. These are also moments when misinterpretation, bias and delay can be introduced, particularly when services are under pressure or under-resourced [1]. However, specific processes targeting these transfer points can produce economic and clinical benefits [2, 3]. This is an area where digital approaches may be helpful [4], especially those using natural language processing (NLP) [5, 6] and fair data re-use [7].

In UK mental health services, a key transfer point is between primary care (general practice) and secondary care mental health services (SMH, including community and hospital services) [8]. Each of these referrals are reviewed within community mental health teams (CMHTs) or specialty services in mental health and discussed in a multi-disciplinary team meeting alongside a review of the EHR. EHRs contain unstructured free text data, are often extensive, and it takes time to assess them to inform decisions. The referral process involves time and costs: in the UK initial assessments arising from new referrals to SMH services cost £326 million in 2018–2019 [9].

In addition, triage processes often lack transparency to patients and referrers, and delays can result from ‘referral bouncing’, from triaging multiple times and from inconsistency and disagreements between teams [10]. This can also result in a ‘hidden waiting list’ [11], with significant impacts for patients, carers and health and social care services. For example, in a survey study of 535 British adults with a mental illness, the impacts of worsening mental health reported included needing to use emergency services or a crisis line for support, and financial, employment and relationship difficulties [12]. This may be particularly relevant in the treatment pathways for clinical depression. Depressive disorder is managed initially in primary care, with referral to secondary care if there is no or minimal response after one or more first-line interventions. However, pressures on secondary care services mean that only those patients most at risk (usually assessed as an immediate suicide risk) are accepted by secondary care. Consequently, people with ‘treatment-resistant’ depression (TRD)—or the broader concept of ‘difficult-to-treat’ depression (DTD)—may remain in the gap between primary and secondary care, often cycling through repeated referrals without receiving timely or effective care [13, 14].

In the UK there has been a recent emphasis on providing more specialist services to people with DTD, as they experience more prolonged symptoms, higher healthcare use and poorer outcomes than other subtypes of depression. Addressing the referral complexities of patients meeting criteria for DTD also closely aligns with the priorities of the UK’s Mental Health Mission [15], a new national initiative aimed at transforming mental health services by improving accessibility, integration, and quality of care from primary through to secondary care.

NLP-based tools could help clinicians in primary and secondary care by identifying people who might have, or be at-risk of, DTD. A previous study has shown the feasibility of identifying patients with DTD from electronic health records in this way [16]. Clinicians already use predictive models such as QRISK routinely to identify people at risk of cardiovascular morbidity [17], and similarly a DTD-caseness tool could assist clinicians in making referral and treatment decisions. In previous proof-of-concept work [4], we showed that large language models could be used to construct digital triage assistance for ‘specialist’ teams (i.e., those focused on, for example, intellectual disability, eating disorders and early intervention for psychosis) by exploiting semantic similarities between patients’ EHR data *without* explicitly identifying a patient’s caseness for a particular disorder/phenotype.

### Study objectives

The CHRONOSIG (CHRONOlogical SIGnature) project aims to improve the referral and triage process by applying machine learning (ML) technology to the information in EHR data [18]. Engagement is a key area in digital approaches [19] and therefore the current project used a focus group methodology to ascertain the views of people with lived experience. The topics discussed were on the use of CHRONOSIG and similar digital approaches to support decision making in triaging referrals in mental health, specifically to identify TRD and DTD, and the potential benefits and disadvantages of such an approach.

## Methods

### The CHRONOSIG project

In the UK, each patient’s historical EHR data captures episodes of care under mental health services. CHRONOSIG uses NLP techniques to provide a patient’s longitudinal signature (or ‘fingerprint’) capturing their history, signs, symptoms and presenting difficulties and then learns associations between these signatures and triage decisions. This approach supports the strategic priorities of the Topol review [20] and the NHS Long Term Plan [21] which emphasise the use of EHRs to improve and personalise care for individuals. The CHRONOSIG project aims to deliver technology to support triage via a clinical decision support tool (CDST) that takes a patient’s referral documents and existing medical notes (when available) and delivers a suggested triage outcome to assist SMH services.

### Focus group

The aim of the focus group was to provide more detailed information about patient views of the potential use of a CDST in mental health, using CHRONOSIG as the specific example. Further details are available in Appendix 1 in the supplementary material, but the broad topic prompts included a short opening section with introductions before prompts to discuss CDSTs in general, differences between using these in physical and mental health disorders and the use of CHRONOSIG specifically in DTD. In particular, the focus group participants were invited to consider the current use of AI tools in clinical consultations (e.g., using a QRISK score for predicting risk of heart attack/stroke) before discussing their usefulness for treatment of DTD. Finally, there was a focus on potential benefits or harms from use in the mental health setting, including possible impacts on the patient pathway across different services.

The focus group was conducted on 25th September, 2024. Participants were recruited by convenience sampling from existing lived experience groups recruited to the CHRONOSIG project and the Mental Health Research for Innovation Centre (https://mric.uk), part of the UK Mental Health Mission and the groups were based in Oxford and Liverpool. Members of these two groups were recruited explicitly for their lived experience of having navigated mental health services, either themselves or as a carer. All participants were co-researchers who received compensation for their time participating in patient and public involvement research in both projects. Participants received participant information via email with an invitation to participate. They gave email confirmation of their consent to participate before the group, with the opportunity to ask further questions if needed. The participant information is contained in Appendix 2 and included a summary of the project with explanations and a range of questions which they were asked to consider before they participated in the group.

The focus group lasted for 1 h and 30 min and was facilitated by JHH supported by DWJ and HP using a semi-structured topic guide (Appendix 1). The group was conducted remotely, digitally recorded using Microsoft Teams, automatically transcribed and checked manually. The data were analysed thematically and managed using the Framework method [22], with double coding of transcripts by two researchers (KAS and HP) to ensure consistency. To reduce any potential bias in extracting and interpreting the results, these two researchers (KAS and HP) had no previous involvement in CHRONOSIG and came from two different research groups. One attended the focus group and the other was separate, analysing only the transcript of the group. Once the two researchers had coded and categorised the data within the Framework matrix, the research team discussed any emerging findings to aid interpretation and explore and develop themes relating to participants’ views and experiences. The COREQ (Consolidated Criteria for Reporting Qualitative Research) guidelines [23] were used to report the qualitative results (Appendix 3). All co-authors read and approved the final paper before submission, but to minimise bias the CHRONOSIG group did not make any material changes to the results or conclusions, which were analysed and generated by the research team.

## Results

A total of 16 participants (9/16 female, 56%) were included. 14/16 provided their age which ranged from 35–71 years (median (IQR) 52.5 (48–66) and of the 15 who provided data on ethnicity, the majority identified as White British (13/15, 87%), with the remaining 2 participants identifying as ‘White Other’ and ‘Mixed’. The main themes, with illustrative quotes arising from the framework analysis of the content of the focus groups are summarized in Table 1, and the full framework analysis is in Appendix 4.

**Table 1:** Main themes with example quotes from the focus group

Theme/Subtheme	Relevant quotes
*Theme 1: The complexity of mental health needs assessments*	
Depression is multifactorial and difficult to capture	*The factors …… causing depression seem so diverse and particular. I wondered how neatly itemised they could be?*
Assessments need to be holistic	*Unless you can see the holistic, the full view of the person that you’re treating, you may well go down a rabbit hole that really doesn’t help the situation*
Assessments need to consider background and childhood	*You know we all have had situations, I suspect, with trauma, in childhood*
Assessments need to consider social factors	*The social determinants of things that make us where we are in later life*
Mental and physical health are usually interlinked and overlap	*The two are linked and inter-linked. People have a level of health which includes physical health and mental health*
*Theme 2: Challenges in the current mental health system*	
Incomplete notes	*Everybody has their own patient story, and I’m concerned that it’s not all on record*
Notes are in different places	*In my case the documentation on my mental health journey is held by many different places, and sometimes the GP doesn’t have that data*
Lack of continuity in healthcare provision	*I am not likely to see the same GP*
Lack of collaboration between teams	*I think [it is] working in collaboration. So, with GP and then referral to the specialist clinicians, ……working together in a collaboration [with] effective communication and then going through to the specialist*
Long waits for treatment and virtual waiting lists	*I don’t think clinicians do that. …. I think they just dish out medication, and they send the patient on their way, and the patient is waiting a long time for therapy*
*Theme 3: General challenges of using a computer/AI based tool for risk prediction and clinical decision support*	
The output could only be as good as the data entered	*[I] distrust AI because of"garbage in, garbage out”* *It’s down to obviously the GP, the specialist, whoever you see. But my concern is these things can get missed… they’re not picked up*
The efficacy of the tool depends on the data it is trained on	*How could the tool be trained …… without somebody knowing what factors are, and what percentages are?*
Concerns around the ‘explainability’ of AI	*I would also want to know what factors the tool had taken into account in making its findings …..The clinician may not know how the tool has reached its conclusion*
Potential negative outcomes	*You could end up traumatising a patient really by saying in 10 years that you’ve got 32% chance of having a heart attack or stroke…. We have to be aware of the patient how they’re going to be feeling when they’re told this*
Potential benefits	*I would see it as a constructive thing to work with hopefully, …. as a call to good change rather than it being a negative thing*
*Theme 4: Differences and similarities in using a computer-based prediction tool in mental health vs a risk prediction tool in physical health such as Q-Risk*	
Differences	*Estimating someone someone’s risk of a heart attack or a stroke…. seems to be more factual and probably easier to estimate than estimating someone’s risk of depression, which is more based on patient factors*
Both are interrelated	*One could affect the other. So, depression can affect your chance of a heart attack…. So, I think they’re all completely linked*
Similarities	*For me, there’s no difference, absolutely no difference …. I’m someone with multiple long-term conditions, but I’m also at the intersectionality of having multiple, long term mental health conditions, and my risk of death from the mental health conditions is equal to the risk from physical health conditions*
*Theme 5: Possible benefits and harms in using a clinical prediction tool in mental health*	
* Potential harms*	*The harm is how it is used, simple as that, how it is used and the risk of losing the integral humanity of the clinician patient interface* *The information fed in may be incorrect thereby giving a false prediction*
* Potential benefits*	
• General benefits	*I would be happy to use any tool available to get to the bottom of my problem. I think technology is amazing* *This is such an important tool to add to what is in place for difficult to treat depression, which is so resistant to treatment*
• Improved accuracy of prediction	*AI is better at prediction than human beings*
• Patient preference	*Some patients may prefer AI and engage with healthcare services earlier, i.e., increased prevention and earlier diagnosis*
• Improve treatment options	*What this tool should be doing is encouraging people, the clinicians, to identify a plan going forward to help the individual with their mental conditions* *It would just mean that I have to be prepared for maybe a different kind of treatment …. so it might mean more intensive therapy or a different kind of therapy*
• Improve waiting times and efficiency	*The benefit would be about time. That’s a major factor* *But now you’re sitting on a waiting list for a year. So for me…. you can identify my problem, or, potentially what could help me*
• Improve identification of specialist needs	*[Identifying that] I would need more specialist support to get the right treatment* *I think that if it encourages treatments that hadn’t previously been considered, or provides mechanisms or routes through for additional support that haven’t previously been given, that’s a good thing*
*Theme 6: Factors to consider in the future use of a clinical decision tool in mental health*	
Inclusivity	*Accessibility of information- re language, ethnicity, learning style, neurodiversity etc are important*
An addition not a replacement for the clinical consultation	*It shouldn’t replace the human element of treatment*
Empathy remains a key factor	*Effective communication is very important. and if you have effective communication with your GP, the trust comes. If …. I’m seeing the same GP, I will trust him*

In total, 6 main themes emerged from the analysis. These were:

1. the complexity of mental health needs assessments;
2. challenges in the current mental health system;
3. general challenges of using a computer/artificial intelligence (AI) based tool for risk prediction and clinical decision support;
4. differences and similarities in using a computer-based prediction tool in mental health vs. a risk prediction tool in physical health such as QRISK;
5. possible benefits and harms in using a clinical prediction tool such as CHRONOSIG in mental health, specifically in DTD;
6. factors to consider in using this in future.

In theme 1 (the complexity of mental health needs assessments), many participants commented on the complexity of adequately assessing mental health needs, and the necessity of taking a holistic view encompassing background, childhood, social and physical as well as specific mental health factors. Many participants commented that they felt that mental and physical health issues were often interlinked and affected each other.

Theme 2 (challenges in the current mental health system) built on this complexity, with participants identifying their views of the challenges in the current mental health services. With respect to clinical decision making, many participants identified difficulties with accuracy and incomplete medical notes. This was compounded as several participants commented that GPs and family physicians seem short of time and they perceived a lack of continuity. Participants identified that therefore the notes and written referral process may be even more vulnerable to omissions, errors and potential delays. Several participants raised their concerns about long waits for specialist treatment and being ‘stuck’ between services.

Theme 3 (general challenges of using an AI based tool) explored the general approach of a digital or AI based CDST. Participants raised issues about the need to provide the tool with accurate data (from medical notes in the EHR) and to train the tool on the relevant and appropriately diverse datasets. Discussions of risk in general were felt to have both advantages and potential harms and many commented that they felt that risk tools need to be used in the context of a high-quality clinical consultation.

Theme 4 (differences and similarities in using a computer-based prediction tool in mental health vs physical health) explored the possible additional complexities of using a CDST such as CHRONOSIG in mental health specifically. Many participants commented they felt that DTD seemed to them much harder to predict with accuracy than physical health conditions such as heart attack or stroke, but several felt they should be regarded in the same way and are often interlinked.

Themes 5 and 6 explored possible harms and benefits and considerations for the future. Possible harms were raised by a few participants, mainly focussed on the potential for over reliance on the CDST at the detriment of the face-to-face clinical assessment. However, overall, participants were positive about the CDST and identified many more potential benefits, including improved decision making, improved accuracy, reduced bias, in particular for underrepresented communities [24], improved patient preference and widening the range of treatment options. A number of participants identified the potential benefits in terms of time and efficiency in referrals to specialist care. Many participants also felt there were also potential benefits in more accurately identifying specialist treatment needs and facilitating these referrals earlier with reduced waiting times. In future uses (Theme 6) participants identified their assessments of key factors to consider, including appropriate diversity and inclusivity and specific training needs for clinicians. The group were unanimous in expressing the need for the CDST tool not to be used as standalone instrument, but as an integrated part of a clinical consultation which includes empathy as part of a strong therapeutic relationship.

## Discussion

### Principal findings

In this study, we sought the views of patients, those with lived experience and participants in the use of an AI assisted CDST specifically focussed on identifying risk and improving the referral process for those with DTD. Whilst previous studies have investigated the use of CDSTs in mental healthcare (mainly around risk assessment, for example Golden 2024 [25]), they have focussed on clinician feedback and mental healthcare in general [26].

Through the focus group, participants quickly engaged with the concepts of CHRONOSIG and were able to comment on the benefits and disadvantages of using such tools in healthcare in general. The specific use in mental healthcare triage and referral in DTD was a novel area and they gave valuable insights. Attitudes were generally positive. Participants had already identified delays, errors and confusion in the referral process and expressed positive views on the ability of the CHRONOSIG tool to help to improve waiting times and time spent between services (often referred to in the literature as the ‘hidden waiting list’) as a particular area of concern.

### Comparison with previous studies

Previous studies have looked at digital and AI approaches to support various aspects of decision-making in mental health, although approaches have been variable. For example, a recent scoping review found only 12 relevant studies, together covering different aspects of decision-making support: diagnostic and predictive AI, treatment selection AI, and self-help AI [26]. Of note all the studies identified focussed on clinician feedback rather than patient or lived experience views or shared decision making. In the included studies, clinicians generally showed a high level of trust in AI systems aiding decision making, with the amount of use of the AI systems in treatment selection being associated in one study with the amount of clinician’s knowledge of e.g. machine learning methods and their role in AI [27]. This is key, as in the adoption of a digital tool, clinicians also have an important part to play in engagement [19] and training programmes for clinicians in digital approaches in the mental health setting are already established [28].

However, whilst clinicians have a key role, shared decision making equally involves the person with mental illness, and patient feedback on AI assisted CDSTs in mental health has been highlighted as a target for future research [26, 29]. In this study we focussed on exactly this area. Future research could also include primary and secondary care clinicians and managers, as collaboration with a multidisciplinary team including clinicians, machine learning engineers, and patients using the AI (as well as wider systems such as hospital and general practice providers) is key to achieving effective implementation [30].

In the focus group, participants identified without specific prompts some of the key issues which have been highlighted in previous studies. For example: issues of ‘explainability’ (broadly, the ability of a human to *understand* how or why an AI tool delivered a given output), which can reduce user acceptance and decision-making quality [31, 32], issues of using non-representative or biased data in training AI tools [33] and the need for adjustment for patient-physician communication when using the AI approaches [34].

Themes 5 and 6 from our results (including the potential for over-reliance on AI-driven CDSTs and provision to safeguard against AI technology being used for efficiency gains at the expense of relational, face-to-face models of care) speak directly to newer models of biomedical ethics and socio-technical practice. This is pertinent given the EU AI Act’s emphasis on a risk-based approach to regulation [35] and the contested GDPR ‘right to explanation’ in automated decision making [36]. Some popular reporting [37] describes a tension between ethical, governed AI and hindering the pace of technological development (the ‘regulation *vs* innovation’ dichotomy, [38]. However, others have argued that legislation alone is too nebulous to be practically operationalised [39] and advance a new model of “embedded ethics” where ethicists work alongside technologists, developers and those with lived-experience throughout the development, testing and subsequent deployment of AI. While this provides an oversight function, it does not mechanistically change how AI systems are trained and used; in this vein, [40] argue for ‘human-in-the-loop’ methods as a default for ensuring accountability when developing AI systems and in the deployment context, [41] argue for considering AI as ‘boosting’ human performance rather than replacing a human in a process.

The focus group participants also raised a number of specific points concerning the patient’s journey through different care delivery teams. They highlighted the current potential for delays and confusion in the referral process, concerns about incomplete or fragmented notes and the lack of continuity of clinician across repeated consultations. Whilst identifying some potential disadvantages, many participants were positive about the impact that an AI-assisted CDST could make in improving accuracy and decision making, reducing waiting times (including hidden waits), reducing bias in triage decisions, improving options for patient preference and widening the range of treatment options. This area is relevant in the context of today’s mental health care services. In many countries there are shortfalls in mental health care provision with increasing waits in both recorded and unrecorded waiting lists. For example, in the UK, in December 2024, a total of 2.0 million people in England were in contact with SMH services and during this month 407,255 new referrals were received by SMH services and 2.02 million care contacts were attended [8]. There is also a ‘hidden waiting list’ [11] with significant impacts: in a report from the Royal College of Psychiatrists in the UK, nearly a quarter of mental health patients (23%) waited more than 12 weeks to start treatment, 43% said that the wait between initial referral and second appointment had caused their mental health to worsen, and 78% of those on the ‘hidden waiting list’ reported that they had to access emergency services or a crisis line in the absence of mental health support [12]. Thus, any intervention which can streamline and improve accuracy in referral processes could have profound effects on health and social care services and the wellbeing of patients and carers.

### Strengths and limitations

As with any focus group the ideas expressed reflect the views of the specific focus group members. We recorded participant characteristics in terms of age, gender and ethnicity. However, the majority of the sample identified as White and although there was a range of ages, younger and much older adults were not represented. In addition, it might also have been helpful to explore other characteristics such as prior experience/familiarity with technology and the nature of their prior experience in the health care system. However, this was a relatively large group with a mix of age and sex. We chose a larger group to enable a range of voices to be heard, but to address issues that may arise with larger groups (such as dominance of a subgroup of voices) we used a variety of approaches including using a facilitator with lived experience, providing pre-reading, information and questions to consider, and extending the length to 90 min to allow more nuanced discussion. To mitigate any potential bias, the analysis was performed by two independent researchers, one who had observed the focus group and the other who had not been involved. The study used a qualitative approach to identify the key themes arising from the focus group discussion and to direct further research in this area. Building on this study, a quantitative or mixed methods approach (for example, Smith 2023 [42]) could be used to identify the relative importance of these themes from the lived experience perspective.

## Conclusions

Using observational data from a large collection of the secondary care mental health EHR in the UK alongside state-of-the art neural network algorithms for NLP, we and our collaborators have together demonstrated the feasibility of developing conceptual models capable of capturing a patient’s trajectory from clinical notes [43–46]. In this study, through a focus group process co-researchers with lived experience were consulted on how automation tools for identifying ‘caseness’ (ie meeting criteria for DTD) and system efficiency (digitally assisted triage) from the EHR for a complex mental illness presentation (DTD) might be deployed. We explicitly used an analogy with similar tools in the management of cardiovascular disease (QRISK) to elicit views on how mental and physical health differ in the use of such tools. We focussed on those CDSTs that automatically ingest a patient’s clinical data from EHRs and deliver an output that might result in potential benefits (for example by reducing referrals, or by facilitating more timely access to appropriate care) or potentially cause harms (such as a loss of relational care by reliance on technology). Our findings indicate that people with lived experience offer a cautiously optimistic view on the use of intelligent operational automation tools based on longitudinal patient data from historical EHRs. Just as in the use of QRISK, their perspective was that this could be a useful addition, rather than replacement, to a high-quality clinical consultation. Taking into consideration the specific use-case, the individual disorder and accounting for patients’ and lived experience participants’ concerns, these results suggest that automation could help streamline the process of care in the NHS. This can also be used to help identify those people with mental health issues who have the highest care needs or sub groups who will be most suited for different services and who might be most eligible for individual clinical research trials.

## Supplementary Information

Supplementary Material 1.

## Data Availability

All data generated or analysed during this study are included in this published article (and its supplementary information files).

## Abbreviations

AI: Artificial Intelligence
EHRs: Electronic health records
NLP: Natural language processing
CHRONOSIG: (CHRONOlogical SIGnature) project
ML: Machine learning
SMH: Secondary care mental health services
TRD: Treatment resistant depression
DTD: Difficult-to-treat-depression
CMHTs: Community mental health teams
CDST: Clinical decision support tool
(COREQ) guidelines: Consolidated Criteria for Reporting Qualitative Research

## References

[1] Docherty M, Thornicroft G. Specialist mental health services in England in 2014: overview of funding, access and levels of care. Int J Mental Health Syst. 2015;9(1):34. 10.1186/s13033-015-0023-9 PMC4553216 26322123

[2] Kim S, Chepenik LG. Use of computer modeling to streamline care in a psychiatric emergency room: a case report. Psychiatr Serv. 2020;71:92–5. 31590624 10.1176/appi.ps.201900040

[3] Rycroft-Malone J, Fontenla M, Bick D, Seers K. Protocol-based care: impact on roles and service delivery. J Eval Clin Pract. 2008;14:867–73. 19018920 10.1111/j.1365-2753.2008.01015.x

[4] Taylor N, Kormilitzin A, Lorge I, Nevado-Holgado A, Cipriani A, Joyce DW. Model development for bespoke large language models for digital triage assistance in mental health care. Artif Intell Med. 2024;157:102988. 39383705 10.1016/j.artmed.2024.102988

[5] Kormilitzin A, Vaci N, Liu Q, Nevado-Holgado A. Med7: a transferable clinical natural language processing model for electronic health records. Artif Intell Med. 2021;118:102086. 34412834 10.1016/j.artmed.2021.102086

[6] Vaci N, Liu Q, Kormilitzin A, De Crescenzo F, Kurtulmus A, Harvey J, et al. Natural language processing for structuring clinical text data on depression using UK-CRIS. BMJ Ment Health. 2020;23(1):21–6. 10.1136/ebmental-2019-300134 PMC10231554 32046989

[7] Joyce DW, Kormilitzin A, Hamer-Hunt J, McKee KR, Tomasev N. Defining acceptable data collection and reuse standards for queer artificial intelligence research in mental health: protocol for the online PARQAIR-MH Delphi study. BMJ Open. 2024;14(3):e079105. 38490661 10.1136/bmjopen-2023-079105 PMC10946350

[8] NHS Digital. Mental health services monthly statistics, performance, December 2024. 2025. https://digital.nhs.uk/data-and-information/publications/statistical/mental-health-services-monthlystatistics/performance-december-2024.

[9] NHS England. National Cost Collection 2019. NHS England and NHS Improvement. 2020. https://www.england.nhs.uk/wp-content/uploads/2020/08/1_-_NCC_Report_FINAL_002.pdf.

[10] Chew-Graham C, Slade M, Montana C, Stewart M, Gask L. A qualitative study of referral to community mental health teams in the UK:exploring the rhetoric and the reality. BMC Health Serv Res. 2007;7(1):117. 10.1186/1472-6963-7-117 PMC1971054 17651489

[11] RCPsych. Two-fifths of patients waiting for mental health treatment forcedto resort to emergency or crisis services. 2020. https://www.rcpsych.ac.uk/news-and-features/latest-news/detail/2020/10/06/two-fifths-of-patients-waiting-for-mental-health-treatment-forced-to-resort-to-emergency-or-crisis-services.

[12] RCPsych. Hidden waits force more than three quarters of mental health patients to seek help from emergency services. 2022. https://www.rcpsych.ac.uk/news-and-features/latest-news/detail/2022/10/10/hidden-waits-force-more-than-three-quarters-of-mental-health-patients-to-seek-help-from-emergency-services#:~:text=Nearly%20a%20quarter%20of%20mental,for%20World%20Mental%20Health%20Day.

[13] Paganin W, Signorini S, Sciarretta A. Difficult-to-treat depression Scoping review. Clin Neuropsychiatry. 2023;20(3):173. 37522111 10.36131/cnfioritieditore20230302 PMC10375274

[14] Parker G. A revisionist model for treatment-resistant and difficult-to-treat depression. Aust N Z J Psychiatry. 2024;58(6):460–6. 38539283 10.1177/00048674241240600 PMC11128139

[15] Mental Health Mission, NIHR 2023. https://www.nihr.ac.uk/news/ps427-million-funding-boost-mental-health-research.

[16] Costa T, Menzat B, Engelthaler T, et al. The burden associated with, and management of, difficult-to-treat depression in patients under specialist psychiatric care in the United Kingdom. J Psychopharmacol. 2022;36(5):545–56. 10.1177/02698811221090628. 35506640 10.1177/02698811221090628 PMC9112623

[17] Hippisley-Cox J, Coupland C, Brindle P. Development and validation of QRISK3 risk prediction algorithms to estimate future risk of cardiovascular disease: prospective cohort study. BMJ. 2017;357:j2099. 10.1136/bmj.j2099. 28536104 10.1136/bmj.j2099 PMC5441081

[18] Joyce DW, Kormilitzin A, Hamer-Hunt J, James A, Nevado-Holgado A, Cipriani A. CHRONOSIG: digital triage for secondary mental healthcare using natural language processing-rationale and protocol. medRxiv. 2021:2021–11.

[19] Smith KA, Ward T, Lambe S, Ostinelli EG, Blease C, Gant T, et al. Engagement and attrition in digital mental health: current challenges and potential solutions. npj Digit Med. 2025;8(1):398. 40604240 10.1038/s41746-025-01778-w PMC12223045

[20] The Topol Review. Preparing the healthcare workforce to deliver the digital future. 2019. https://topol.hee.nhs.uk/.

[21] UK Government. Fit for the future: 10 year health plan for England. 2025. https://www.gov.uk/government/publications/10-year-health-plan-for-england-fit-for-the-future.

[22] Gale NK, Heath G, Cameron E, Rashid S, Redwood S. Using the framework method for the analysis of qualitative data in multi-disciplinary health research. BMC Med Res Methodol. 2013;13(1):117. 10.1186/1471-2288-13-117. 24047204 10.1186/1471-2288-13-117 PMC3848812

[23] Tong A, Sainsbury P, Craig J. Consolidated criteria for reporting qualitative research (COREQ): a 32-item checklist for interviews and focus groups. Int J Qual Health Care. 2007;19(6):349–57. 10.1093/intqhc/mzm042. 17872937 10.1093/intqhc/mzm042

[24] Kormilitzin A, Tomasev N, McKee KR, Joyce DW. A participatory initiative to include LGBT+ voices in AI for mental health. Nat Med. 2023;29(1):10–1. 36631594 10.1038/s41591-022-02137-y PMC7614833

[25] Golden G, Popescu C, Israel S, Perlman K, Armstrong C, Fratila R, et al. Applying artificial intelligence to clinical decision support in mental health: what have we learned? Health Policy Technol. 2024;13(2):100844.

[26] Auf H, Svedberg P, Nygren J, Nair M, Lundgren LE. The use of AI in mental health services to support decision-making: scoping review. J Med Internet Res. 2025;24(27):e63548. 10.2196/63548 PMC11806275 39854710

[27] Jacobs M, Pradier MF, McCoy TH Jr, Perlis RH, Doshi-Velez F, Gajos KZ. How machine-learning recommendations influence clinician treatment selections: the example of the antidepressant selection. Transl Psychiatry. 2021;11(1):108. 10.1038/s41398-021-01224-x. Medline: 33542191. 33542191 10.1038/s41398-021-01224-x PMC7862671

[28] Smith K, Torous J, Cipriani A. Teaching telepsychiatry skills: building on the lessons of the COVID-19 pandemic to enhance mental health care in the future. JMIR Ment Health. 2022;9(10):e37939. 35358948 10.2196/37939 PMC9617186

[29] Knyahnytskyi O, Eadie J, Asadpour K, Stephenson C, Yang M, Reshetukha T, Moi C, Barrett T, Hicks M, Gutierrez G, Kumar A. Perceptions and insights: A qualitative assessment of an AI-assisted psychiatric triage system implemented in an outpatient hospital setting. medRxiv. 2025:2025–03. 10.1177/20552076251384835 PMC12536140 41122425

[30] Nair M, Svedberg P, Larsson I, Nygren JM. A comprehensive overview of barriers and strategies for AI implementation in healthcare: mixed-method design. PLoS ONE. 2024;19(8):e0305949. 10.1371/journal.pone.0305949. 39121051 10.1371/journal.pone.0305949 PMC11315296

[31] Higgins O, Short BL, Chalup SK, Wilson RL. Artificial intelligence (AI) and machine learning (ML) based decision support systems in mental health: an integrative review. Int J Ment Health Nurs. 2023;32(4):966–78. 36744684 10.1111/inm.13114

[32] Joyce DW, Kormilitzin A, Smith KA, Cipriani A. Explainable artificial intelligence for mental health through transparency and interpretability for understandability. NPJ Digit Med. 2023;6:6. 10.1038/s41746-023-00751-9. 36653524 10.1038/s41746-023-00751-9 PMC9849399

[33] Ferrara E. The butterfly effect in artificial intelligence systems: implications for AI bias and fairness. Mach Learn Appl. 2024;15:100525. 10.1016/j.mlwa.2024.100525.

[34] Ferrario A, Gloeckler S, Biller-Andorno N. AI knows best? Avoiding the traps of paternalism and other pitfalls of AI-based patient preference prediction. J Med Ethics. 2023;49(3):185–6. 10.1136/jme-2023-108945. 36788037 10.1136/jme-2023-108945

[35] The European Parliament and The Council Of The European Union, 13 June 2024. The EU Artificial Intelligence Act. 2024. https://artificialintelligenceact.eu/the-act/.

[36] Wachter S, Mittelstadt B, Floridi L. Why a right to explanation of automated decision-making does not exist in the general data protection regulation. Int Data Privacy Law. 2017;7(2):76–99.

[37] Lagercrantz O. Europe’s AI Act stumbles out of the gate. The Center for European Policy Analysis; 2025. https://cepa.org/article/europes-ai-act-stumbles-out-of-the-gate/.

[38] Csernatoni R. The EU’s AI power play: between deregulation and innovation. Carnegie Endowment for International Peace. 2025. https://carnegieendowment.org/research/2025/05/the-eus-ai-power-play-between-deregulation-and-innovation?lang=en.

[39] McLennan S, Fiske A, Tigard D, Müller R, Haddadin S, Buyx A. Embedded ethics: a proposal for integrating ethics into the development of medical AI. BMC Med Ethics. 2022;23(1):6. 10.1186/s12910-022-00746-3 PMC8793193 35081955

[40] Chandler C, Foltz PW, Elvevåg B. Improving the applicability of AI for psychiatric applications through human-in-the-loop methodologies. Schizophr Bullet. 2022;48(5):949–57. 10.1093/schbul/sbac038 PMC9434423 35639561

[41] Herzog SM, Franklin M. Boosting human competences with interpretable and explainable artificial intelligence. Decision. 2024;11(4):493–510.

[42] Smith KA, Ostinelli EG, Ede R, Allard L, Thomson M, Hewitt K, et al. Assessing the impact of evidence-based mental health guidance during the COVID-19 pandemic: systematic review and qualitative evaluation. JMIR Ment Health. 2023;10(1):e52901. 38133912 10.2196/52901 PMC10760515

[43] Liu Q, Vaci N, Koychev I, Kormilitzin A, Li Z, Cipriani A, et al. Personalised treatment for cognitive impairment in dementia: development and validation of an artificial intelligence model. BMC Med. 2022;20(1):45. 35101059 10.1186/s12916-022-02250-2 PMC8805393

[44] Senior M, Burghart M, Yu R, Kormilitzin A, Liu Q, Vaci N, et al. Identifying predictors of suicide in severe mental illness: a feasibility study of a clinical prediction rule (Ox ford M ental I llness and S uicide Tool or OxMIS). Front Psychiatry. 2020;11:268. 32351413 10.3389/fpsyt.2020.00268 PMC7175991

[45] Taylor N, Schofield D, Kormilitzin A, Joyce DW, Nevado-Holgado A. Developing healthcare language model embedding spaces. Artif Intell Med. 2024;158:103009. 39500238 10.1016/j.artmed.2024.103009

[46] Vaci N, Koychev I, Kim CH, Kormilitzin A, Liu Q, Lucas C, et al. Real-world effectiveness, its predictors and onset of action of cholinesterase inhibitors and memantine in dementia: retrospective health record study. Br J Psychiatry. 2021;218(5):261–7. 32713359 10.1192/bjp.2020.136

