# Supplementary material for: AI assisted triage of UK patients in mental health care services: a qualitative focus group study of patients’ attitudes
Source: BMC Psychiatry. 2026 Jan 13;26:35. doi: 10.1186/s12888-025-07329-7 (PMC12801542; doi:10.1186/s12888-025-07329-7)
Supplement: Supplementary file 1 — Supplementary Material 1. [file 12888_2025_7329_MOESM1_ESM.docx]

**APPENDIX**

**Contents**

Appendix 1 Topic guide …………………………………………………………………………………………………………………… 2

Appendix 2 Participant information and questions for consideration………………………………………………..3

Appendix 3 Consolidated criteria for reporting qualitative studies (COREQ): 32-item checklist...........6

Appendix 4 Framework analysis of the focus group transcripts.………………………………………………………12

1. **Topic guide for the focus group**

The following questions are intended as guidance questions for the focus group and will be developed as the focus group progresses.

**Openers**

Introductions and housekeeping rules:

- permission to record
- timekeeping
- guidance on putting hand up
- using the chat
- taking breaks

**Focus group topic guide questions (each introduced with a slide and explanation**)

- If you visited a healthcare provider for problems with your mood, how would you feel if they used a computer-based decision support tool (CDST) to discuss your difficulties with you?
- When estimating someone’s risk of a health problem, should depression and heart attack/stroke be viewed differently?
- If your GP explained that a CDST (such as QRISK) estimates you have a 32% chance of having a heart attack or stroke in the next ten years, what does that mean to you? [Accompanying diagram showing example of QRISK2]
- If your psychiatrist explained that a CDST (such as CHRONOSIG/the DTD algorithm) estimates you have a number of factors that might make your mood problem more difficult to treat, what does that mean to you? [Accompanying diagram showing example output of proposed DTD tool]
- Thinking about either heart attack / stroke or difficult to treat depression:
  - Can you identify any benefits to the use of CDSTs?
  - Are there any harms from the use of CDSTs?

**Closing discussion**

Next plans:

- use of the data
- publication plans
- dissemination amongst the focus group members

1. **Participant information**

**Title: Stakeholder Consultation on Clinical Decision Assistance using AI in Mental Healthcare**

**Context and Example:** For many decades, researchers have looked for ways to improve the reliability of how human clinicians make decisions using computers and technology. This is particularly true in recent years because of the explosion of interest in Artificial Intelligence.

Clinicians sometimes use software to help them decide what to do in certain, usually complex, cases. These “clinical decision support tools” (CDSTs) are usually pieces of software that use algorithms to take inputs (usually, data describing some factors about the patient) and output something that the clinician hopefully uses to help improve the patient’s condition and quality of life.

An example is the QRisk calculator, used by general practitioners (GPs). QRisk takes as its input data like the patient’s blood pressure, cholesterol level, whether or not they smoke and if they have other conditions such as migraines, rheumatoid arthritis and so on. Given this data, QRisk then outputs a percentage or probability that the patient will have either a heart attack or a stroke in the next 10 years. If QRisk outputs a value of between 10-20%, the patient is deemed at medium risk of having a heart attack or stroke, and the GP can then discuss with the patient options for treatment that would be expected to *lower* that risk.

The QRisk software is sometimes built into the GPs electronic health record system (e.g. the patient’s electronic medical notes or record), for convenience and to encourage its use. The web-based version of the current QRisk CDST is available online here: <https://qrisk.org/>

**Our Project:** People who have depression sometimes recover with the first or second treatment; for example, some people have counselling, cognitive behaviour therapy and/or some people use medications. However, many people with depression do not improve after the first, second and sometimes many more treatments. This is due to many and varied reasons. It is difficult to ‘tease apart’ the reasons why people have not improved after recommended ‘first-line’ treatments and may require specialist assessment and treatments. We call this “difficult-to-treat” depression. We think there is value in developing tools like QRisk, especially to help identify people who have difficult-to-treat depression.

**What is Difficult-to-Treat Depression (DTD)?** Difficult-to-Treat Depression (DTD) refers to a type of depression that continues to have a significant impact on a person's life despite receiving standard treatments. Understanding DTD involves looking at various factors related to the patient, the nature of the illness, and the treatments used, to understand why standard approaches might not be working.

**We would like you to consider this scenario:** our CDST takes inputs describing the patient’s difficulties and symptoms of depression and estimates the chances that the person will have “difficult-to-treat” depression.

**More Detail on Our Project:** We are developing a clinical decision support tool (CDST) to help identify patients who might have DTD. Our proposal is that the CDST will use information from a patient’s Electronic Health Record (EHR). Unlike heart attacks and strokes that are the focus of QRisk, the data required for difficult-to-treat depression is not as straight-forward as entering the patient’s blood pressure or ticking a box to clarify if they have migraines or not. Instead, we need to use algorithms that can ‘read’ data written in natural language (rather than being numerical, like someone’s blood pressure or cholesterol level).

**For example:** a 43-year-old patient has had a difficult childhood and early adult life, and first had symptoms of depression aged 17; they have tried two separate periods of cognitive behavioural therapy and more recently, used a number of different medications. However, they still experience symptoms of depression that seem not to improve. Our fictional CDST takes this data as input and gives an estimate of how likely the person will have “difficult to treat” depression, e.g. the CDST outputs that there is a 73% chance (that is, it is really quite likely) they have this condition. Their GP could then discuss with the patient different treatment options, that might include seeing a doctor, psychologist or other healthcare team who specialise in helping people with difficult to treat depression.

**How Does Our Tool Work?** We need some technology that can read and locate the important details from text in the electronic health record that we understand can incline someone to experience difficult to treat depression. If we had such an algorithm, it might ‘pick out’ the highlighted phrases from the patient’s medical records like the example below:

“A 43 year old patient has had a difficult childhood and early adult life, and first had symptoms of depression aged 17; they have tried two separate periods of cognitive behavioural therapy and more recently, used a number of different medications. However, they still experience symptoms of depression that seem not to improve.”

In the above example, our imagined algorithm has picked out:

- Blue phrases are those data that tell us about the patient’s life history or facts about them now
- Light red phrases are those that describe the history of the patient’s symptoms
- Light orange phrases are those that describe medication treatments and if they have (or have not) worked
- Light purple phrases are those describing psychological treatments and if they have (or have not) worked

After extracting these data from the text describing the patient’s difficulties, our proposed CDST will then tell the clinician and patient what these factors are, and provide some numerical or graphical indication of how likely the patient is to have difficult-to-treat depression.

**Why Your Input Matters:** We are hosting a Patient and Public Involvement and Engagement (PPIE) meeting to gather your feedback and to understand how people would view the CDST that we’ve described above. We want to ensure that the development and use of this new tool are ethical, acceptable and beneficial to the people who might have depression and clinicians who are tasked with trying to help them. Your feedback will help us refine the tool and ensure it meets your needs.

**Questions for the Audience:**

**Understanding and Concerns:**

- 1. Do you understand what Difficult-to-Treat Depression (DTD) is?
  2. Do you think our CDST may be appropriate for helping identify DTD?
  3. What are your concerns about a tool that analyses your health records to identify DTD?

**Communication and Transparency:**

- 1. How important is it to you to be informed about the results from CDSTs? For example, considering how QRisk is used for heart attacks and strokes and then thinking about our proposed difficult-to-treat depression tool?
  2. What information would you like to receive about how the tool works?

**Impact and Benefits:**

- 1. How useful do you think the proposed CDST tool may be for helping patients and clinicians improve treatments and management?
  2. What potential benefits or unintended harms do you see in using this tool for identifying and managing DTD?

**Trust and Reliability:**

- 1. What would help you trust the results provided by this tool?
  2. Are there specific features or assurances you would need to feel confident in its use?

**Privacy and Data Use:**

- 1. What safeguards would you want to see to make you feel comfortable about your health data being used to develop the tool?

Your feedback is crucial in shaping the development of this tool. Thank you for your participation and valuable insights.

1. **Consolidated criteria for reporting qualitative studies (COREQ): 32-item checklist**

| **No** | **Item** | **Guide questions/description** | **Response** |
| --- | --- | --- | --- |
| **Domain 1: Research team and reflexivity** |  |  |  |
| Personal Characteristics |  |  |  |
| 1. | Interviewer/facilitator | Which author/s conducted the interview or focus group? | JHH – lead facilitator  Assisted by DWJ for time keeping and HP for note-taking. |
| 2. | Credentials | What were the researcher's credentials? *E.g. PhD, MD* | BSc (JHH), MBBS / MD (DWJ) and Ph.D. (DWJ, HP) |
| 3. | Occupation | What was their occupation at the time of the study? | PPIE co-researcher,  Research Scientist and  Psychiatrist |
| 4. | Gender | Was the researcher male or female? | Lead facilitator – female  Assisting - male |
| 5. | Experience and training | What experience or training did the researcher have? | All had focus group training (via their respective university training programmes). JHH has facilitated focus groups for xx years; DWJ, 10 years and AK xx years. |
| Relationship with participants |  |  |  |
| 6. | Relationship established | Was a relationship established prior to study commencement? | Yes – all participants were members of either Oxford Health BRC’s PPIE group, the CHRONOSIG project’s PPIE group or M-RIC’s PPIE group. |
| 7. | Participant knowledge of the interviewer | What did the participants know about the researcher? e*.g. personal goals, reasons for doing the research* | JHH was unknown to the participants; DWJ was known to 3 participants from previous PPIE events around technology in mental healthcare |
| 8. | Interviewer characteristics | What characteristics were reported about the interviewer/facilitator? e.g. *Bias, assumptions, reasons and interests in the research topic* | JHH reported her career history as a member of the Oxford BRC PPIE group having worked on a diverse range of research involving patients. DWJ did not describe his research interests except to identify as one of the researchers on CHRONOSIG. |
| **Domain 2: study design** |  |  |  |
| Theoretical framework |  |  |  |
| 9. | Methodological orientation and Theory | What methodological orientation was stated to underpin the study? *e.g. grounded theory, discourse analysis, ethnography, phenomenology, content analysis* | The data were analysed thematically and managed using the Framework method (Gale 2013). |
| Participant selection |  |  |  |
| 10. | Sampling | How were participants selected? *e.g. purposive, convenience, consecutive, snowball* | Purposive convenience sampling of a population who have previously self-selected as paid lived-experience co-researchers on CHRONOSIG, the wider Oxford Health BRC PPIE group and the MRIC PPIE group. |
| 11. | Method of approach | How were participants approached? e*.g. face-to-face, telephone, mail, email* | Email approach describing the focus group and inviting participation. Pre-reading and suggested questions circulated in advance. |
| 12. | Sample size | How many participants were in the study? | N=16 |
| 13. | Non-participation | How many people refused to participate or dropped out? Reasons? | One participant responded initially and then dropped out, no reason was given. |
| Setting |  |  |  |
| 14. | Setting of data collection | Where was the data collected? e*.g. home, clinic, workplace* | The group was conducted remotely, digitally recorded using Microsoft Teams, automatically transcribed and checked manually |
| 15. | Presence of non-participants | Was anyone else present besides the participants and researchers? | HP attended the meeting and took contemporaneous notes |
| 16. | Description of sample | What are the important characteristics of the sample? *e.g. demographic data, date* | See results section |
| Data collection |  |  |  |
| 17. | Interview guide | Were questions, prompts, guides provided by the authors? Was it pilot tested? | See Appendix 1, topic guide. |
| 18. | Repeat interviews | Were repeat interviews carried out? If yes, how many? | No |
| 19. | Audio/visual recording | Did the research use audio or visual recording to collect the data? | The group was conducted remotely, digitally recorded using Microsoft Teams, automatically transcribed and checked manually |
| 20. | Field notes | Were field notes made during and/or after the interview or focus group? | Yes, by HP |
| 21. | Duration | What was the duration of the interviews or focus group? | 1.5 hours |
| 22. | Data saturation | Was data saturation discussed? | No, as only one focus group was conducted |
| 23. | Transcripts returned | Were transcripts returned to participants for comment and/or correction? | No |
| **Domain 3: analysis and findings** |  |  |  |
| Data analysis |  |  |  |
| 24. | Number of data coders | How many data coders coded the data? | Two, KAS and HP |
| 25. | Description of the coding tree | Did authors provide a description of the coding tree? | Yes, the framework is described in the results section and the full framework analysis is in Appendix 4. |
| 26. | Derivation of themes | Were themes identified in advance or derived from the data? | From the data |
| 27. | Software | What software, if applicable, was used to manage the data? | Data were encoded into an excel spreadsheet and then managed manually |
| 28. | Participant checking | Did participants provide feedback on the findings? | Yes, the paper was circulated to all co authors prior to submission, including the CHRONOSIG group. All co-authors read and approved the final paper before submission, but to minimise bias the CHRONOSIG group did not make any material changes to the results or conclusions, which were analysed and generated by the research team. |
| Reporting |  |  |  |
| 29. | Quotations presented | Were participant quotations presented to illustrate the themes / findings? Was each quotation identified? e*.g. participant number* | Yes  Yes |
| 30. | Data and findings consistent | Was there consistency between the data presented and the findings? | Yes |
| 31. | Clarity of major themes | Were major themes clearly presented in the findings? | Yes |
| 32. | Clarity of minor themes | Is there a description of diverse cases or discussion of minor themes? | Yes |

**Framework analysis of the focus group transcripts**

Ages are self-identified and coded as: U29 (29 and under), 30s, 40s, 50s, 60s, 70s, PNS (prefer not to say)

Ethnicity is self-identified and coded as: WB (White British), WO (White other), MBI (Mixed (British/Iraqi)), ABP (Asian British (Pakistani)).

Sex is self-identified and coded as F (female), M (male)

**THEME 1: THE COMPLEXITY OF MENTAL HEALTH ASSESSMENTS**

Many of the participants used the group to reflect on their experiences of mental health needs assessments and their views on the complexity of providing a full and accurate assessment incorporating all of the person’s relevant experiences:

*‘The factors behind causing depression seem so diverse and particular. I wondered how neatly itemised they could be?’* [M, 60s, WB]

Some participants noted the importance of a holistic assessment incorporating diverse factors:

*‘Unless you can see the holistic, the full view of the person that you're treating, you may well go down a rabbit hole that really doesn't help the* *situation‘* [M, 70s, WB]

Several participants commented on the importance of including family background and childhood experiences:

*‘Things that make us where we are in later life, you know we all have had situations, I suspect, with trauma, in childhood’* [M, 70s, WB]

*‘I think it also depends on whether it runs in your family as it does in mine; I know it's looming on the horizon.’* [F, PNS, WO]

Social factors were also raised by several participants as an important consideration in assessments:

*‘The social determinants of things make us where we are in later life’* [M, 70s, WB]

Most participants commented on how mental and physical health are usually interlinked and conditions can overlap:

*‘People have multiple diagnoses’* [M, 40s, MBI]

*‘People with depression are more likely to have CVD [cardiovascular disease] amid other conditions.’* [F, PNS, WO]

*‘The two are linked and inter-linked. People have a level of health which includes physical health and mental health.’* [M, 60s, WB]

**THEME 2: CHALLENGES IN THE CURRENT MENTAL HEALTH SYSTEM**

The majority of participants noted that they felt that currently, mental health assessments are often hampered by incomplete notes:

‘*NHS notes are not always accurate’* [F, PNS, WO]

*‘Everybody has their own patient story, and I'm concerned that it's not all on record’* [F, 70s, WB]

*‘I recently did ask for my medical records…..so from birth to now, and there was so much information [that] wasn't in it. There was a lot of information that was incorrect’* [F, 50s, WB]

Some participants also raised their concern that notes were often held in different places:

*‘In my case the documentation on my mental health journey is held by many different places, and sometimes the GP doesn't have that data’* [F, 60s, WB]

Participants also noted that they felt that doctors were often short of time:

*‘It's down to obviously the GP, the specialist, whoever you see. But my concern is these things can get missed.’* [F, 50s, WB]

They also noted they were concerned that they did not have continuity of GP provision:

*‘I am not likely to see the same GP’* [F, 60s, WB]

One participant highlighted what they felt was the ideal clinical pathway:

*I think [it is] working in collaboration. So, with the GP and then referral to the specialist clinicians, ……working together in a collaboration [with] effective communication and then going through to the specialist.* [F, 40s, ABP]

However, several participants raised their concerns about long waits for specialist treatment, and being stuck on a ‘virtual waiting list’

*‘What I'd want the patient to be able to do is to know exactly what treatment options they've got in a very short period of time, and that would be access to therapy or to certain medications, and also to be told about the medications, what effect they will have on the patient with their depression. I don't think clinicians do that. I don't think they do it at all. I think they just dish out medication, and they send the patient on their way, and the patient is waiting a long time for therapy’* [M, 40s, MBI]

*‘GPs only have 10 minute slots to see you. By their own admission they are pill givers because they have to react to a condition and have to try and find a cure there and then. So, they always go down the route of prescribing a drug or a referral to another team, and then there is a wait before you can actually see consultants in the secondary sector.’* [M, 70s, WB]

*‘But now you're sitting on a waiting list for a year. So, for me it's like, either, you know, you can identify my problem, or, you know potentially what could help me. But I'm not able to access that.’* [F, 50s, WB]

**THEME 3: GENERAL CHALLENGES OF USING A DIGITAL/AI BASED CDST FOR RISK PREDICTION AND CLINICAL DECISION SUPPORT**

The group raised several areas of challenge they identified in the use of any digital/AI (artificial intelligence) based CDST in healthcare. Many participants identified that the output of the CDST could only be as good as the data which was entered into it:

*‘[I] distrust AI because of "garbage in, garbage out".’* [F, PNS, WO]

*‘Whether that tool has all the information it needed to actually be a flag [of risk] …… in the right way.’* [F, 70s, WB]

Some highlighted concerns that inaccuracies could arise if the inputted data was incomplete

*‘It's down to obviously the GP, the specialist, whoever you see. But my concern is these things can get missed. And then, obviously, they're not picked up’* [F, 50s, WB]

One participant explained that they had had an experience where this had occurred:

*‘For example, I have had various* *QRISK tests ….but only recently I was asked about use of antipsychotics to find that that data had not been factored into the percentage risk’* [F, 60s, WB]

Some noted that the CDST trains on data sets and the effectiveness of the CDST depends on the diversity and relevance of these:

*‘How could the tool be trained …... without somebody knowing what the factors are, and what the percentages are? ‘* [F, 60s, WB]

Many participants raised concerns that any digital CDST needs a diverse range of participants in its training:

*‘These people are neurotypical people, not neurodivergent people. So, within your AI informed approach I would suggest that that would be a massive stumbling block that could either misdiagnose, mistreat, and potentially harm that individual unintentionally.’* [M, 40s, WB]

Some raised their concerns that any such computer-based CDST (such as QRISK) might be too general in its approach:

*‘I personally know QRISK. I've had what they refer to as a health MOT. I think it's too simplistic…in my case it diagnosed me as obese when in fact, I'm quite fit. In fact, muscle is heavier than fat, so although at my age the weight would have suggested I was obese, in fact, I'm very fit.’* [M, 70s, WB]

Most of the participants expressed their concern about the explainability of the digital CDST in assessing risk:

*‘I would also want to know what factors the tool had taken into account in making its findings so I would be disappointed not to be able to have that answered, …..the clinician may not know how the tool has reached its conclusion’* [F, 60s, WB]

*‘The doctor wouldn't know all the different factors the AI is using...how would the doctor know that the information the tool needs to work effectively has been asked by the doctor?’* [F, 60s, WB]

One participant was concerned about information security:

*‘Is anybody thinking of threats to information being taken? ..... So, let's say, Russia decides to take whatever they wish within that realm. That's certainly something that needs to be considered. Obviously, we've got multi factorial identification, but it's not enough.’* [M, 40s, WB]

The group noted negative and positive outcomes from discussing risk (using the example provided of a 32% risk of heart attack/stroke in the next 10 years with the QRISK tool).

Negative outcomes suggested included:

*‘I think it would create a lot of anxiety’* [M, 40s, MBI]

*‘You could end up traumatizing a patient really by saying in 10 years that you've got 32% chance of having a heart attack or stroke. So, I think we have to be aware of ethics, and we have to be aware of the patient how they're going to be feeling when they're told this.’* [M, 40s, MBI]

Positive outcomes were also raised by several participants:

*Very much for me it depends on where the patient is in terms of age for a start, how you'd react in terms of your age, but also your situation in life, because, turning it on its head, it would be telling them that they've got a 68% chance of not having a heart attack in the next 10 years, which for some people in the right situation in life would be a positive thing’* [F, 70s, WB]

*‘I think, as a tool it's fantastic. But I think percentages can confuse people. How do you actually read that? How do you really understand on a human level what 32% chance means?’* [M, 60s, WB]

*‘For me it means that it's not necessarily that I will have a heart attack or stroke. I understand that in 10 years my health will probably degrade, will get worse. and I would try to see how I can improve the outcome to be in the 68% of people who don't have that heart attack or stroke in the next 10 years’* [F, 30s, WO]

*‘I would see it as a constructive thing to work with hopefully, you know, as a call to good change rather than it being a negative thing, and just sending me into a spiral of panic’* [F, 60s, WB]

Some participants also noted that they did not feel convinced yet of the evidence for risk tools in general:

*‘For me, a key supplementary question - this CDST is based upon the PH based QRISK tool, so is there evidence that QRISK is having a measurable positive effect on public health? There would seem to be lessons to be learned from this’* [M, 60s, WB]

**THEME 4: POSSIBLE DIFFERENCES AND SIMILARITIES IN USING A COMPUTER BASED PREDICTION TOOL IN MENTAL HEALTH (VS A RISK PREDICTION TOOL IN PHYSICAL HEALTH SUCH AS QRISK)**

Many of the participants highlighted differences they perceived for a CDST in mental health:

*‘Physical health discussion is much easier than mental health for sharing the story and for our clinicians to diagnose’* [F, 40s, ABP]

*‘Heart attack and stroke factors are more precise and clearly known and can be keyed in by a clinician when the tools made. But, ....with this tool the depression factors are much more nebulous‘* [F, 60s, WB]

*‘Estimating someone's risk of a heart attack or a stroke….seems to be more factual and probably easier to estimate than estimating someone's risk of depression which is more based on patient factors. ...So I do think, yeah, that they should be viewed differently’* [F, 30s, WO]

*‘I think it's quite difficult to predict, difficult to treat depression. I've heard of people who've had really severe depression. They've been hospitalized, and they've been told it will come back, and it doesn't, and I think probably a lot of it is to do with your situation.’* [F, PNS, WO]

Several participants highlighted that they felt they were interrelated:

*‘One could affect the other. So, depression can affect your chance of a heart attack because …. it does things to your blood pressure and … the food you eat and the exercise you get. So, I think they're all completely linked’* [F, 50s, WB]

*‘Physical conditions and mental conditions are inextricably linked’* [M, 70s, WB]

Participants also felt there were similarities:

*‘For me, there's no difference, absolutely no difference …. I'm someone with multiple long-term conditions, but I'm also at the intersectionality of having multiple, long term mental health conditions, and my risk of death from the mental health conditions is equal to the risk from physical health conditions’* [F, 60s, WB]

**THEME 5: POSSIBLE BENEFITS AND HARMS IN USING A CLINICAL PREDICTION TOOL IN MENTAL HEALTH**

Many members of the group were able to contribute their view of potential harms in a risk tool in mental health. Some expressed concerns that a digital risk tool might detract from the empathy of the therapeutic relationship:

*‘I might be slightly concerned….can a computer really answer this question in the way that a human being who has empathy and compassion could answer it? And is it taking away from the doctor's understanding of the condition?’* [F, 40s, WB]

*‘The harm is how it is used, simple as that, how it is used and the risk of losing the integral humanity of the clinician patient interface’* [M, 60s, WB]

*‘I worry that the medical professional, the GP might rely less on their face-to-face observations, and even relegate them or shelve them or dismiss them in contrast to what they've been told by the tool because of the time constraints, so they might be encouraged to do that, despite any misgivings they might be hankering at the back of their mind’* [F, 70s, WB]

*‘Harms: for me [it’s when it’s] not applied in the right way [with] no training, empathy or validation and understanding of the patient’* [M, 40s, MBI]

Participants also expressed concern that data might be incorrect, with negative consequences:

*‘The information fed in may be incorrect thereby giving a false prediction’* [F, PNS, WO]

One participant was concerned that by identifying risk, other treatment options might be excluded:

*‘If told that information by a doctor my query would be: what can be done and what treatment choices I might have? And what would govern which would be offered? I'd hope that would be offered in my area, and also that this diagnosis wouldn't mean I wasn't 'allowed' to go for mainstream regular treatment?’* [F, 60s, WB]

Many participants highlighted what they felt would also be potential benefits:

*‘I'd be comfortable with it. We're all getting used to the use of AI in our day-to-day lives and I personally support its use in healthcare’* [M, 40s, WB]

*‘I would be happy to use any tool available to get to the bottom of my problem. I think technology is amazing’* [F, 30s, WO]

Some participants highlighted that they felt positive about any additions to decision making in mental health:

*‘This is such an important tool to add to what is in place re difficult to treat depression, which is so resistant to treatment’* [F, 60s, WB]

*‘It would cause me anxiety, but if they delivered it in a sensitive and supportive way, I would be keen to know how to prevent the condition happening or getting worse’* [F, 50s, WB]

*‘Sometimes I find it better to know the truth so I know what I’m dealing with….you can sometimes deal with it better’* [F, 50s, WB]

Most participants felt that a tool like this might improve accuracy of prediction:

*‘Using this tool [would] allow the clinician to observe potential red flags which would probably be missed, due to the time pressures during the GP appointments. They don't really have the time to go through the records. I think this is very useful’* [F, 30s, WO]

*‘AI is better at prediction than human beings’* [F, PNS, WO]

One participant noted that they felt that the tool might be less biased in mental health than human clinicians:

*‘As we know, many doctors kind of treat us differently’* [F, 50s, WB]

Another noted that for some patients a computer-based tool might be preferable:

*‘Some patients may prefer AI and engage with healthcare services earlier, ie. increased prevention and earlier diagnosis’* [M, 40s, WB]

Many participants noted that they felt the tool would be useful if it produced a change in action. One change identified was to increase the range of treatment options:

*‘What this tool should be doing is encouraging people, the clinicians, to identify a plan going forward to help the individual with their mental conditions’* [M, 70s, WB]

Participants also identified that they felt there would be advantages in terms of time and efficiency in referral to specialist care:

*‘I think it's a very useful tool, as the GPs, most of the time, they don't have the time to go through the records, to see a pattern or … to notice any issues. So this could potentially be missed. So this tool with having accurate records in place, and the right guidance on how to use it. I think it would be very useful’* [F, 30s, WO]

*‘Benefits absolutely because it pulls together such a plethora of information from so many data sets that doctors would not be familiar with or have access to’* [M, 60s, WB]

*‘Yeah, the benefit would be about time. That's a major factor’* [F, 50s, WB]

*‘[There are] benefits timewise. Management is a really good one, and it's cost effective’* [F, 40s, ABP]

Participants also felt there might be benefits in identifying specialist treatment needs:

*‘I might be encouraged that it might be another string to the bow, as it were, of trying to work out what's going on and what might help me, and focus all our minds, especially if I was feeling quite distressed. As long as it was suggested in a constructive way that didn’t mean I couldn't chat to the doctor in general terms’* [F, 60s, WB]

*‘Benefits - to identify in a timely way a previously disregarded issue with difficult to treat depression’* [F, 60s, WB]

*‘I think that if it encourages treatments that hadn't previously been considered, or provides mechanisms or routes through for additional support that hasn't previously been given, that's a good thing’* [F, 70s, WB]

*‘So, I think any tool that allows this kind of conversation to be prompted around mental health and previous mental health and to identify and facilitate those kind of conversations is actually really very useful’* [F, 60s, WB]

Some participants also noted they felt this tool could reduce waiting times and facilitate referral to specialist care:

*‘But now you're sitting on a waiting list for a year. So for me….. this can identify my problem, or, potentially what could help me’* [F, 50s, WB]

*‘[Identifying that] I would need more specialist support to get the right treatment’* [F, 30s, WO]

*‘But it would just mean that I have to be prepared for maybe a different kind of treatment …..so it might mean more intensive therapy or a different kind of therapy’* [F, PNS, WO]

*‘I think there's no doubt that a tool that will increase our knowledge is a hugely beneficial thing’* [F, 70s, WB]

*‘It might indicate that I would probably need further assessment and maybe more specialist support. I imagine difficult to treat depression ……could possibly get under control with the right treatments. It is not necessarily impossible to treat it. It's just I would need more specialist support to get the right treatment’* [F, 30s, WO]

**THEME 6: FACTORS TO CONSIDER IN THE USE OF A CLINICAL DECISION TOOL IN MENTAL HEALTH**

Participants felt that a number of specific factors needed to be considered. These included inclusivity:

*‘Accessibility of information- re language /ethnicity/learning style/neurodiversity etc are important’* [F, 60s, WB]

*‘It might be a BAME community where English is their second language. It could be a problem. It may be there are barriers, language barriers, cultural barriers. There may be people [who] are elderly who are not used to it, and they are the old school of thought, and they might …..find it difficult’* [F, 40s, ABP]

*‘When somebody's talking about mental health it might be for some communities [that] it is stigmatised because mental health is stigmatised. It's not easy to share the story of personal experience. People may not like to share, and some people just feel reluctant [because] of the barriers’* [F, 40s, ABP]

*‘You've got to be careful about making [it] too simplistic, an algorithm that ignores the complexity of life’* [M, 70s, WB]

Several participants identified training needs:

*‘The training for the person who's using the tool…. would be vital to make sure the person was using it appropriately, and not just kind of switching off and pushing buttons when you're trying to sit and have a conversation about your health’* [F, 50s, WB]

All participants expressed that they felt it was essential that the tool was used in addition to the clinical consultation:

*‘It shouldn't replace the human element of treatment’* [F, PNS, WO]

*‘And I understand this tool would not diagnose. It is just it could potentially flag up something that is relevant to my problem’* [F, 30s, WO]

Empathy was identified by all participants as a key element in use of the tool:

*‘Effective communication is very important. and if you have effective communication with your GP, the trust comes. If ….. I'm seeing the same GP, I will trust him’* [F, 40s, ABP]

*‘The bond that we have with our doctors? If they're compassionate and kind. ....It's the communication between the doctor, the clinician, and the patient that's the most important, and recognising …… the way that you communicate conditions is paramount to the reception by the patient’* [M, 70s, WB]

*‘How they deliver that information to the patient that they're sitting with, to be able to have that open discussion so that they can work together in collaboration to go forward, because not only …..is this a point of transition for the patient, maybe having to face up to a diagnosis, but a particular look at their condition’* [F, 60s, WB]

One participant summarised their view of the ideal role of the tool within the therapeutic relationship:

*‘It is a call to action, but what action and how this is addressed depends so much on the therapeutic relationship - doctors’ empathy and competence cannot be written out of the equation’* [M, 60s, WB]
